# Scientific Careers

**Published:** 1876-08

**Authors:** 


Scientific Careers.—M. Fremy, of Paris, the eminent chemist, thinks that he has discovered a means for winning back to the study of science those who now desert it for more lucrative pursuits. He proposes to create for savans a modest progressive pension comparable to that offered by the State to the soldier or the civil engineer. According to this project, the scientific career is to comprise five grades. The savans of the 5th grade are to receive pensions of $600 per annum; those of the 4th grade, $1000 per annum; those of the 3d grade, $1600; those of the 2d, $2000; and finally, those who reach the first grade, $3000. A special jury is to be appointed to determine the capacity of the young savans, and to propose them for advancement, according to the amount of real service that they have rendered to science. M. Fremy's plan is ingenious and captivating, but unfortunately, in conceiving it he has given no thought to the many and apparently insuperable objections that present themselves to it.—*Med. Record.*

